# Task-shifting eye care to ophthalmic community health officers (OCHO) in Sierra Leone: A qualitative study

**DOI:** 10.7189/jogh.11.07001

**Published:** 2021-03-10

**Authors:** Vladimir Pente, Stevens Bechange, Emma Jolley, Patrick Tobi, Anne Roca, Anna Ruddock, Nancy Smart, Kolawole Ogundimu, Matthew Vandy, Elena Schmidt

**Affiliations:** 1 Sightsavers, Yaoundé, Cameroon Country Office; 2 Sightsavers, Freetown, Sierra Leone Country Office; 3 Sightsavers, Haywards Heath, UK; 4 Middlesex University, London, UK; 5 Sightsavers, Abuja, Nigeria Country Office, Nigeria; 6 Ministry of Health and Sanitation, Freetown, Sierra Leone

## Abstract

**Background:**

Preventing visual impairment due to avoidable causes has been a long-standing global priority. Of all blindness in Sierra Leone, 91.5% is estimated to be avoidable and 58.2% treatable, however there are only 6 ophthalmologists for the whole country. Task-shifting has been suggested as a strategy to address this issue and a training intervention was developed to create a cadre of community-based staff known as Ophthalmic Community Health Officers (OCHOs). This qualitative study aimed to explore the experiences of OCHOs, their relationship with other eye health workers, and how they interact with the wider health system, in order to provide recommendations for the design and delivery of future task shifting strategies.

**Methods:**

Between April and May 2018, we conducted semi-structured interviews with 42 participants including: OCHOs (n = 13), traditional ophthalmic staff (n = 17) and other stakeholders from the districts (n = 6), training institution staff (n = 4) and MOH headquarters (n = 2). We identified participants using purposive sampling. Interviews were audio-recorded, transcribed, and thematically analysed. We draw largely on in-depth interviews but complement the analysis with evidence from a document review.

**Results:**

In Sierra Leone, the roll-out of the OCHO programme presented a mixed picture. OCHOs participating in the study expressed a strong commitment to their new role. However, policy changes proposed to clearly demarcate roles and responsibilities and institutionalise the cadre in the civil service were not implemented, resulting in the posting of some staff at an inappropriate level, dissatisfaction with the OCHO certification, and lack of opportunities for advancement and training. These challenges reflect structural weaknesses in the health system that undermine a cohesive implementation of eye health initiatives at the primary health care level in Sierra Leone.

**Conclusions:**

Task-shifting has the potential to improve provision in under-resourced specialities such as eye health. However, the success of this approach will be contingent upon the development of a robust and supportive health policy environment.

Human resources are an essential component of global health systems, but the world is facing a chronic shortage of trained health workers.[1] The situation is particularly alarming in sub-Saharan Africa (SSA) [2]. Eye care is not an exception to this crisis, with an average of 2.7 ophthalmologists per million population in SSA [3-5], compared to 31 per million globally [6]. Additionally, ophthalmologists in SSA tend to be located in urban centres, leaving large parts of rural communities with no access to eye care [7,8]. The availability and distribution of the eye health workforce has been highlighted as a priority for action in the recent World Report on Vision [1,9,10].

In response to the growing demand for health care and persistent shortages of health workers, the World Health Organisation (WHO) has encouraged the adoption of a task-shifting strategy [1,11], which makes use of available human resources by delegating specific tasks from a higher level cadre to a lower level one [2,11]. Successful examples of task-shifting in eye care come from Neglected Tropical Disease (NTD) programmes, where community volunteers distribute drugs to prevent onchocerciasis [12] and nurses perform trichiasis surgeries [11,13]. The use of primary and community health workers in other aspects of eye care has been under debate. Those in favour of the approach [14] argue that lower level health care workers are better placed to raise community awareness, find asymptomatic patients and treat simple eye conditions, preventing complications and saving patient and provider time and costs. However, there is limited real life evidence on the opportunities and challenges of task-shifting eye care tasks within wider primary health systems [14,15].

Sierra Leone is one of the poorest countries in the world, [16] and presents some of the worst health indicators [17-19]. The prevalence of blindness among people aged 50+ years is estimated at 5.9% with over 91% of the burden considered avoidable [20,21]. The latest Eye Health System Assessment conducted in 2013 shows densities of 1.8 ophthalmologists/cataract surgeons and 6.8 ophthalmic nurses per million population [20], below the recommended regional targets of 4 and 10 per million, respectively. About 80% of ophthalmologists, 50% of cataract surgeons, and 39% of ophthalmic nurses are located in the capital city, serving 16% of the country’s population [22].

The Community Health Officer (CHO) cadre was established in Sierra Leone in the 1980s to provide frontline primary health care in rural communities [23]. CHOs receive three years of basic medical training [23]. They are salaried civil servants within the Ministry of Health and Sanitation (MoHS) and posted at the lowest tier of the health care system, in Peripheral Healthcare Units (PHU), where they are the first point of contact for patients, especially at Community Health Centres (CHCs) [23,24]. CHOs provide a range of preventive and clinical health care services and they work under the immediate professional and administrative supervision of the District Medical Officer (DMO) [24] who is often in charge of CHCs with a catchment area of 10 000 to 30 000 people [23]. At the grassroot level, the health system extension and basic service delivery is provided by Community Health Workers (CHWs). They serve as a link between CHCs and the communities and deliver health education, screening and referrals [25].

In 2011, the MoHS in collaboration with Njala University and other stakeholders, launched a programme to train CHOs in clinical sub-specialties, as recommended by WHO [26]. The training was residential and delivered by Njala University. Initial specialisations included minor surgeries and obstetrics, followed by ophthalmic care and mental health. The programme for ophthalmic CHOs (OCHOs) began in 2012, and aims to strengthen primary eye care services by shifting tasks from mid-level ophthalmic cadres working at the secondary level to CHOs [27]. Programme participants must have had the initial CHO qualification and two years’ experience in the government sector. OCHOs are trained in basic ophthalmology over 18 months and are expected to return to their community health units to deliver basic eye care services alongside other health care responsibilities **(****Figure 1****)**. No refresher training or on-site mentorship was planned as part of the training programme. The key OCHO competencies include diagnosing and managing common eye conditions, prescribing and dispensing simple ophthalmic drugs, performing minor eye surgeries, and organising outreach services [27]. The training modules also include algorithms for patient management to ensure that patients with complex eye conditions requiring more advanced ophthalmic care are identified and referred.

**Figure 1:**
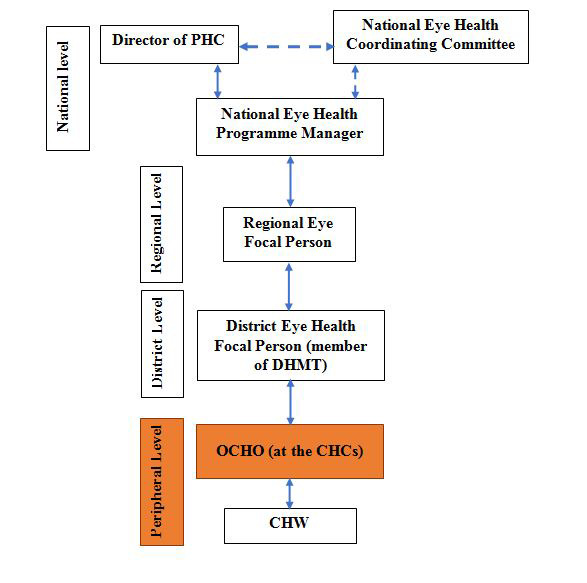
Communication pathways for Sierra Leone National Eye Health Programme. Source: Adapted from the National Eye Health Policy Sierra Leone 2018-2022.

The OCHO programme has not been evaluated and little is known about its implementation. This study explored the experiences of OCHOs trained under this new programme. It focused specifically on the OCHOs’ perception of the training, their post-qualification deployment, their relationships with colleagues and their interactions with the wider health system. It is envisaged that the insights from this study will be useful for the design and delivery of future similar task-shifting programmes in Sierra Leone and more widely.

## METHODS

### Study design and sampling

The study used a qualitative design with semi-structured interviews to collect data. All OCHOs trained and operational by January 2018 were invited to participate. Other stakeholders involved in the programme, including primary health care officials and health managers, teaching staff and other health care workers were also purposefully selected and invited to participate.

Interviews were supplemented by documentary analysis of MoHS policies, the OCHO curriculum and the regulatory framework. However, we did not have access to the hospital records and cannot say whether the task-shifting has resulted in lower number of patients with simple eye care conditions presenting at the secondary level.

### Data collection and analysis

All interviews were conducted in English by four researchers (males-VP, PT, MV and one female, NS) in April- May 2018. Interviews were conducted in a place convenient to the participants and lasted between 40 and 60 minutes. Three topic guides were developed with questions relevant to different groups of participants. The OCHOs’ topic guide was focused on the OCHOs’ experiences of their training, the application of new skills, daily work, and how they perceived their role within the health system. The two other topic guides for stakeholders from the MoHS, training institution staff and other health care workers working alongside the OCHOs were focused on the selection process, the legal framework, and the working relationship with OCHO. Interviews were audio recorded and transcribed verbatim.

Data was analysed thematically using NVIVO 11 [28] following six stages: familiarisation with the data; coding; generating, reviewing and defining themes; and writing up. The coding framework was developed after reading the first batch of transcripts. All transcripts were coded independently by three researchers (VP, PT and EJ). A fourth researcher (SB) was involved in discussing discrepancies and reaching a consensus. Data interpretation was validated with a selected number of participants from different stakeholder groups during a workshop.

### Ethical considerations

The study was approved by the Sierra Leone Scientific and Ethics Committee. All participants were informed about the objectives and process of the study. Participation was voluntary and there were no incentives provided in this study. A written informed consent was obtained from all participants. All data were anonymised and kept on password protected computers.

## RESULTS

### Participant characteristics

In total, 42 participants were interviewed (36 males and 6 females). While a balance by sex was initially sought for the sample, the composition of the pool of participants to draw from was found to be largely skewed towards males.

By the time of the study, 16 OCHOs had completed their training and were deployed in government health facilities. All 16 were invited to participate but only 13 (11 males and 2 females) were available. The median age of interviewees was 38 years. Data on the years of experience was available for 12 participants with four each having >5, 2-3 and <2 years of experience. OCHOs were placed across eight out of 11 Sierra Leone districts ranging from 1 to 3 per district. Eight OCHOs were deployed at PHUs, while five were based at district hospitals.

Other study participants included District Medical Officers (n = 6), national level officials (n = 2), other eye care workers (n = 17) and training institution staff (n = 4).

### Motivation for enrolment to the OCHO programme

OCHOs gave three main reasons for enrolling on the programme. First, many expressed a sense of responsibility towards their community. They knew that eye diseases were common but there were no qualified eye care workers in their areas, and adherence to referrals in the community was an issue. As one OCHO explained:

“The reason why I became so interested in the programme was because during the CHO training we do not have much learning, especially about eye care. So, most of the cases we used to refer elsewhere. But sometimes, those cases we refer do not go for referral and they use native herbs instead. So that made me interested so I can take care of minor [cases] and refer them to their appropriate place.” *Male OCHO 11*

For some OCHOs, their interest in eye care stemmed from experiences with poor vision in their family. OCHOs wanted to gain new knowledge and skills to be able to address eye problems that were preventable or treatable:

“Oh yes, I have an aunt who is totally blind, due to glaucoma and she is suffering. We, her family members, are supporting her, but it is difficult for us. She feels alone and for anything she wants or needs, she is unable to do herself and needs to call somebody for help [...] You might find yourself in that position, so that is what actually motivated me to learn more about the eye.” *Female OCHO 9*

Many OCHOs saw the new programme as an opportunity for career progression. Two participants were already qualified in eye care at the ophthalmic nurse level and thought that the training would allow them to specialize further. But most saw it as an opportunity to diversify the range of their clinical skills as CHOs:

“Well I was practicing as CHO, but I wanted to do a further course so that I can upgrade myself and I was interested in the eye service.” *Male OCHO 12*

### Training and post-training professional development

Overall, OCHOs reported enjoying the training and found it comprehensive and informative. Respondents were positive about the duration, content of the curriculum and quality of teaching. These positive impressions however were overshadowed by dissatisfaction with the level of certification, lack of opportunities for further training and uncertainties about their future career. Participants had enrolled in the programme with the understanding that they were to receive training at the higher diploma level. Instead, they were awarded a lower academic qualification at the ordinary diploma level, which was frustrating, as it did not meet expectations:

“Yes […] it was an ordinary diploma and it was embarrassing for us; that was not what we were expecting. They gave us an identification card from the union, which showed a higher diploma and an acceptance letter that was written with a higher diploma; only that when the result came, it was only an ordinary diploma. Many of us felt that we were not graded according to our performance, they just wrote an ordinary pass.” *Male OCHO 12*

Another issue was a lack of refresher training. A number of OCHOs said that a one-off training course provided only limited knowledge about eye diseases. Participants wanted to have opportunities to refresh their skills. This was particularly the case for the OCHOs involved in the Ebola outbreak, who felt out of practice but had no way to refresh their knowledge when they started practicing again:

“[…] in eye cases, you need in-service training because after our training, we were faced with […] Ebola and that prevented us from using our [eye health] skills.” *Male OCHO 2*

Another source of frustration was a lack of clarity about career progression and skills development. The OCHO curriculum had pathways for further professional development, including competencies in community eye health, public health, health education and health management. However, specialisation in eye surgery – an area of interest of many OCHOs – was not included:

“I appreciate the program, but my issue is we were to be trained as cataract surgeons since 2014 but it has not been done.” *Female OCHO 10*

Some respondents noted that the OCHOs who wished to continue their studies in universities in the West Africa region also faced difficulties because their diploma was not recognised outside of Sierra Leone:

“Yes, there are challenges in eye health care as this OCHO course is a national course and eye health care courses are usually being done out of the country. So, some are facing challenges to further their studies outside the country, as some institutions do not recognise the OCHO certificate.” *OCHO coordinator training institution*

### Regulatory framework and remuneration of OCHOs

MoHS officials indicated that the OCHO regulatory document was available, although they themselves had limited knowledge of its content. The general understanding was that the regulatory framework integrated the OCHO cadre competencies within the CHO scope of work. However, in spite of attempts to locate the document, the study team was unable to access it and verify the exact wording. The only available document with a clear description of roles and competencies was the OCHO training curriculum, but this did not include the kind of regulatory information we were looking for.

Study participants identified a number of issues arising from the lack of access to clear regulations for the OCHO cadre. Officers at the district level felt that the absence of a specific document defining the OCHO role meant that the boundaries of their responsibilities were not clearly delineated and there was a risk of OCHOs operating outside the limits of their job.

“That [policy document] needs to be developed because even the [OCHO] cadre itself has challenges, as far as the policies are concerned ... That needs to be …developed. [...] Some of them tend to be enthusiastic and go beyond their limit but that needs constant engagement to give them defined roles and responsibilities in the facility.” *District Medical Officer 5, District level*

Respondents further explained that the government scheme of services document stated that upon graduation, OCHOs should be promoted from grade 5 (CHO) to grade 7. None of the 13 OCHOs participating in the study had been promoted and none had had their salary increased following the training. OCHOs felt undervalued, underpaid, and demotivated, as one OCHO explained:

“I have filled in the forms in 2016 and submitted them to the Ministry for […] promotion and an increment of salary. But up until now, I have not got any feedback, and this demotivates me.” *Male OCHO 3*

Government officials also confirmed that delivering on the promises made to the OCHOs in terms of their career and pay scale was a challenge:

“There is a gap in terms of […] payment of salaries for the work they are doing now because it is a change of status from CHO to OCHO. The documents signed include an increment of salaries and promotion of their cadre because they have done a specialised course [...] As far as I am concerned, […] this has created a scenario wherein most of the trained OCHOs are becoming disinterested […] because they are not paid according to their cadre […] and the salary is low compared to the work they do in the communities.” *CHO Supervisor, MoHS*

### OCHOs’ deployment and responsibilities

Out of 13 OCHOs interviewed, eight were deployed, as intended, at the PHU level. However, five OCHOs worked at the secondary hospital. When asked about the reasons, some participants were unsure and raised the issue of poor coordination between the OCHO training programme and the Ministry structures responsible for staff posting. Others indicated that the reason was understaffing of eye specialist units at secondary facilities, which were not able to receive referrals from the primary level, making the deployment of specialist eye care works at that level impractical.

“They are coming from PHUs. So, they should return to the PHU. [However] Pujehun hospital does not have an eye specialist, the CHO there was trained and was brought to the DHMT [district health management team] to serve as an eye focal person.” *District Medical Officer 4, District level*

In some districts, OCHOs reported being deployed to other roles unrelated to eye care depending on the district needs and shortages of health care staff:

“So, after the Ebola outbreak, they [district authorities] say I’m going to stay here and be a surveillance officer and I was receiving back-up from the hospital. They would call me to work together and I also helped in the nursing training programme. Sometimes, I would end up deviating away from the hospital, because due to your knowledge and performance, they will be giving you more work to do.” *Male OCHO 12*

Managing common eye conditions and dispensing simple eye care medicines were the most common regular tasks reported by OCHOs interviewed. These were also the eye care tasks the OCHOs felt most competent to do. Treatment of eye infections in particular was reported to be most common; eye infections was also the only eye health indicator collected by primary care clinics at the time of the study, although there were plans to introduce more indicators at this level.

Minor eye surgery was the least common procedure reported by OCHOs; nine of the 13 OCHOs had never performed this task. Although many OCHOs said that they wanted to develop minor surgery skills, they did not feel competent enough to perform surgeries at present.

### Infrastructure and resources available to OCHOs

One of the key challenges was the lack of equipment and supplies. It was reported that districts did not have any budgets earmarked specifically for eye health. Although there were discussions at the level of the government to allocate resources for OCHOs through the national eye care plan, the allocations never materialised. As a result, OCHOs had to rely on the support of international donors and iNGOs. Where this support was not available, the equipment and medical supplies were reported to be old and poorly maintained and there were major problems with the procurement of medicines:

“The equipment I am using at the clinic was given to me during the training in the form of a toolbox and this equipment was very archaic and not good for any purpose. To be honest, the clinic is totally without eye equipment and this to some extent slows down the work in the facility.” *Male OCHO 4*

Another important problem was poor uptake of referrals made by OCHOs to secondary facilities. OCHOs explained that many patients could not afford user fees and travel costs associated with hospital referrals and could not access the treatment prescribed:

“Some people cannot afford money to pay and access the big hospital for further treatment and therefore tend to stay at home, thereby exacerbating the problem. Some of them also face difficulty in getting lodging in the big towns, when they are referred there to get treatment, and most of them are not comfortable with the environment in the big towns.” *Male OCHO 3*

Respondents further noted that the current government initiatives to provide free health care did not help patients with eye problems. Community health insurance did not include eye care procedures in the package and the free health care scheme extended only to limited groups of patients, such as pregnant women or children under 5.

“The biggest challenge was when the free health care was introduced, many people coming to the clinic refused to pay […]. They didn’t even know that the free healthcare [only] caters to certain […] people.” *Male OCHO 4*

### Relationships with other colleagues

OCHOs interviewed described complex relationships with their colleagues at the PHU and district levels. On one hand, OCHOs were treated as an authority on all cases related to eyes. OCHOs themselves were very proud to be able to deliver both general and specialist health care:

“It is very cordial, we mostly group together to discuss on cases, at time when they cannot handle things, they call on me. They usually come with problems to me and I direct them.” *Female OCHO 9*

OCHOs were particularly positive about the relationships they had with community health workers, who were a link between them and the local population as the primary cadre responsible for community sensitisation and mobilisation. They used community networks for disseminating information about eye care and identifying patients with eye problems:

“Yes, we get information from CHWs and other stakeholders who give us information concerning eye health conditions in the community. When the CHWs see certain conditions of eye problems they usually call me and notify me through phone calls. I normally tell them to refer the patient to me or in some cases, I move to see the patient at community level.” *Male OCHO 6*

OCHOs were also positive about the support they had from eye care staff working in upper level facilities, to whom they referred patients. They appreciated when more experienced eye care staff spent their time with OCHOs discussing specific eye care cases:

“We meet very often. I refer cases that I cannot handle in the clinic to the government hospital and sometimes, we meet and discuss on how to handle cases of eye conditions and the medication to administer depending on a reported case.” *Male OCHO 1*

On the other hand, a number of OCHOs said that at times, the relationships with other primary care workers were difficult. Some OCHOs reported that their colleagues believed that they could only deal with eye diseases and could not be trusted with other tasks. Other OCHOs pointed to signs of disrespect from their primary care worker colleagues:

“They see you as a specialist in curing eye issues and they become jealous of you and sometimes they become reluctant when I delegate a particular task to them. Sometimes I have to talk to them to work hard, so that they can also be OCHO in the future if they are interested.” *Male OCHO 3*

## DISCUSSION

One of the key benefits expected from task-shifting in specialties with human resource gaps such as eye care is improved access to specialised services at the primary level [1,25]. In Sierra Leone, the OCHO programme was created with that purpose in mind [27]. Shifting some eye care tasks from ophthalmic nurses to CHOs was intended to increase access to eye health services and strengthen the integration of eye care into primary health care [29-31].

In this study, we found that from the perspective of stakeholders on the supply side of eye care, the roll-out of the OCHO programme presented a mixed picture. The programme as designed was appealing to CHOs and its purpose resonated with their perception of their role in the community. The majority of OCHOs participating in our study were eager to take up their new role and help improve access to eye care for their local communities, who they felt acknowledged and valued their role. However, systemic issues arising from insufficiently defined pathways for policy implementation and poor system support undermined the task-shifting programme at two levels.

At the individual level, lack of a clear OCHO policy framework resulted in demotivation and problems with retention of the newly trained staff. In particular, a framework document highlighting competencies, scope of work, regulatory norms and standards, performance indicators and remuneration, which are core to ensure this cadre is effectively integrated, recognised, and sustained within the health system [32], was clearly missing. Previous reports have described the lack of capacity to manage human resources strategically as a weakness at the level of the MoHS [7]. Similar findings have also been reported in other studies in the region, where ambiguity about entitlements, policies and procedures was the main driver of staff dissatisfaction, which negatively impacted the implementation of task-shifting [32-35]. Evidence also suggests that recognition and a defined status within the health system have high influence on health worker retention and motivation [36]. Our study findings suggest that in spite of their specialisation, OCHOs have not been given a higher status within the national system, nor have they been recognised as a health care cadre at the sub-regional level. Without a clear and well communicated policy apparatus, these challenges will be difficult to fix, with implications for the sustainability of the OCHO programme. A systematic review conducted among health care workers in East Africa reported that lack of appreciation and lack of recognition were the main demotivating factors among health workers [37].

At the system level, the slow or insufficient adaptation of other parts of the health system prevented a smooth integration of the new OCHO role in the primary care system. Inadequate procurement of equipment and supplies to perform the job is one example. Another is that although OCHOs were trained to identify a range of eye conditions, the Health Management Information System (HMIS) used at the primary care level had only one eye care indicator. Similar observations were made in other studies in SSA. For example, a study in Tanzania showed that training primary care nurses in eye health did not result in a better access to services for local populations because other parts of the health systems, such as staff supervision, procurement of medical supplies and information systems, have not been adapted to integrate the new tasks [15].

This study had two key limitations. First, we only had two female OCHO participants. This gender imbalance reflects the high male-to-female ratio among those trained as OCHOs under the programme. Studies in other settings have also found gender imbalances among certain medical cadres in LMICs [38], so recruiting more female OCHOs in future programmes should be a priority and future research should include more female OCHOs to reflect more comprehensively OCHOs perspectives and experiences. Second, we did not interview community members or patients in this study and do not know their perceptions of OCHOs and their willingness to take up eye care services at the primary health care level. Some studies in Asia for example, suggest that communities often perceive eye diseases to be too complex to be dealt with by community health workers, which results in low uptake of referrals made by this cadre [39,40]. A study in Nigeria suggested that community health seeking behaviour may differ according to the type of problem perceived [41]. Future studies in Sierra Leone should explore community views on task shifting of eye care to OCHOs.

## CONCLUSION

While there may be operational quick-fixes for some of the issues raised, the consequences of weak policy architecture make clear that there must be considerable investment in a robust policy environment if the primary health care system in Sierra Leone is to be successful. Systemic changes to remedy these limitations are not easy to implement, but until they are, the early promises of the OCHO programmes will remain stunted, together with improvements in access to basic eye care in Sierra Leone, and efforts towards achieving universal health coverage.
